# Supplementary material for: Multiparameter squeezing for optimal quantum enhancements in sensor networks
Source: Nat Commun. 2020 Jul 30;11:3817. doi: 10.1038/s41467-020-17471-3 (PMC7393128; doi:10.1038/s41467-020-17471-3)
Supplement: Supplementary file 1 — Supplementary Information [file 41467_2020_17471_MOESM1_ESM.pdf]

Supplementary Information for
Multiparameter squeezing for optimal quantum enhancements in sensor networks

Gessner, Smerzi, and Pezzè

I. SUPPLEMENTARY NOTE 1: THE MULTIPARAMETER METHOD OF MOMENTS IN THE CENTRAL LIMIT

Here, we derive the multiparameter sensitivity matrix for the method of moments in the central limit. Assuming a large number μ of repeated measurements, the multivariate central limit theorem [1] ensures that the distribution of sample mean values $\bar{\mathbf{X}}^{(\mu)}$ approaches a Gaussian multivariate distribution

$$P(\bar{\mathbf{X}}^{(\mu)}|\theta) = \sqrt{\frac{\mu}{(2\pi)^M \det \Gamma[\hat{\rho}(\theta), \hat{\mathbf{X}}]}} \exp\left(-\frac{\mu}{2}(\bar{\mathbf{X}}^{(\mu)} - \langle\hat{\mathbf{X}}\rangle_{\hat{\rho}(\theta)})^T \Gamma[\hat{\rho}(\theta), \hat{\mathbf{X}}]^{-1} (\bar{\mathbf{X}}^{(\mu)} - \langle\hat{\mathbf{X}}\rangle_{\hat{\rho}(\theta)})\right) \tag{1}$$

with mean $\langle\hat{\mathbf{X}}\rangle_{\hat{\rho}(\theta)}$ and covariance matrix $\Gamma[\hat{\rho}(\theta), \hat{\mathbf{X}}]/\mu$. The conditions $\langle\hat{X}_k\rangle_{\hat{\rho}(\theta)} = \bar{X}_k^{(\mu)}$ identify the multidimensional maximum of Supplementary Equation (1). Hence, the multiparameter method of moments maps to a maximum likelihood estimation of all parameters, which saturates the Cramér-Rao bound [2] and asymptotically in the number of measurements μ leads to the estimator covariance matrix $\Sigma = \Sigma_{\mathrm{mm}}$ with

$$\Sigma_{\mathrm{mm}} = (\mu F_{\mathrm{mm}}[\hat{\rho}(\theta), \hat{\mathbf{X}}])^{-1}. \tag{2}$$

Here, $F_{\mathrm{mm}}[\hat{\rho}(\theta), \hat{\mathbf{X}}]$ is the Fisher information matrix for the distribution in Supplementary Equation (1) (that should not be confused with the Fisher information matrix $F[\hat{\rho}(\theta), \hat{\mathbf{X}}]$),

$$(F_{\mathrm{mm}}[\hat{\rho}(\theta), \hat{\mathbf{X}}])_{kl} = \sum_{\bar{\mathbf{X}}^{(\mu)}} p(\bar{\mathbf{X}}^{(\mu)}|\theta)\left(\frac{\partial}{\partial\theta_k} \log p(\bar{\mathbf{X}}^{(\mu)}|\theta)\right)\left(\frac{\partial}{\partial\theta_l} \log p(\bar{\mathbf{X}}^{(\mu)}|\theta)\right), \tag{3}$$

the sum running over all possible values of $\bar{\mathbf{X}}^{(\mu)}$ and $p(\bar{\mathbf{X}}^{(\mu)}|\theta)$ is the probability to observe the sample mean value $\bar{\mathbf{X}}^{(\mu)}$ given that the parameters take on the values θ. The explicit calculation, see Supplementary Reference [2], gives

$$(F_{\mathrm{mm}}[\hat{\rho}(\theta), \hat{\mathbf{X}}])_{kl} = \mu \left(\frac{\partial\langle\hat{\mathbf{X}}\rangle_{\hat{\rho}(\theta)}}{\partial\theta_k}\right)^T \Gamma[\hat{\rho}(\theta), \hat{\mathbf{X}}]^{-1} \left(\frac{\partial\langle\hat{\mathbf{X}}\rangle_{\hat{\rho}(\theta)}}{\partial\theta_l}\right) + \frac{1}{2}\mathrm{Tr}\left\{\Gamma[\hat{\rho}(\theta), \hat{\mathbf{X}}]^{-1}\left(\frac{\partial}{\partial\theta_k}\Gamma[\hat{\rho}(\theta), \hat{\mathbf{X}}]\right)\Gamma[\hat{\rho}(\theta), \hat{\mathbf{X}}]^{-1}\left(\frac{\partial}{\partial\theta_l}\Gamma[\hat{\rho}(\theta), \hat{\mathbf{X}}]\right)\right\}, \tag{4}$$

where the derivatives of the vectors and matrices are defined element-wise. Since we assume $\mu \gg 1$, the contribution of the first term dominates over the second which thus can be neglected. This yields the result

$$\Sigma = (\mu \mathcal{M}[\hat{\rho}(\theta), \hat{\mathbf{X}}])^{-1}, \tag{5}$$

where $\mathcal{M}[\hat{\rho}(\theta), \hat{\mathbf{X}}]$ is the moment matrix.

II. SUPPLEMENTARY NOTE 2: PROPERTIES OF THE MOMENT MATRIX

The central quantity of interest to characterize (generalized) multiparameter squeezing is the moment matrix, defined by

$$\mathcal{M}[\hat{\rho}(\theta), \hat{\mathbf{X}}] = D[\hat{\rho}(\theta), \hat{\mathbf{X}}]^T \Gamma[\hat{\rho}(\theta), \hat{\mathbf{X}}]^{-1} D[\hat{\rho}(\theta), \hat{\mathbf{X}}], \tag{6}$$

where

$$(D[\hat{\rho}(\theta), \hat{\mathbf{X}}])_{kl} = \frac{\partial\langle\hat{X}_k\rangle_{\hat{\rho}(\theta)}}{\partial\theta_l} \tag{7}$$

and $(\Gamma[\hat{\rho}(\theta), \hat{\mathbf{X}}])_{kl} = \langle\hat{X}_k\hat{X}_l\rangle_{\hat{\rho}(\theta)} - \langle\hat{X}_k\rangle_{\hat{\rho}(\theta)}\langle\hat{X}_l\rangle_{\hat{\rho}(\theta)}$ is the covariance matrix with $\langle\hat{X}\rangle_{\hat{\rho}} = \mathrm{Tr}\{\hat{X}\hat{\rho}\}$. An important special case is given by a unitary phase imprinting evolution, when the moment matrix is given by

$$\mathcal{M}[\hat{\rho}, \hat{\mathbf{H}}, \hat{\mathbf{X}}] = C[\hat{\rho}, \hat{\mathbf{H}}, \hat{\mathbf{X}}]^T\, \Gamma[\hat{\rho}, \hat{\mathbf{X}}]^{-1}\, C[\hat{\rho}, \hat{\mathbf{H}}, \hat{\mathbf{X}}], \tag{8}$$

and $(C[\hat{\rho}, \hat{\mathbf{H}}, \hat{\mathbf{X}}])_{kl} = -i\langle[\hat{X}_k, \hat{H}_l]\rangle_{\hat{\rho}}$ is the commutator matrix. In this section, we prove the main properties of the moment matrix.

A. A matrix-valued Cauchy-Schwarz inequality

We first demonstrate a generalization of the Cauchy-Schwarz inequality to matrices, cf. Supplementary References [3, 4]. We denote by $\mathrm{Mat}(n,m)$ the space of real-valued $n\times m$ matrices.
Lemma. Let $A\in\mathrm{Mat}(p,n)$, $B\in\mathrm{Mat}(p,m)$ and let B^TB be invertible. Then the following matrix inequality holds:

$$A^TA\geq A^TB(B^TB)^{-1}B^TA, \tag{9}$$

and equality is reached if and only if there is some $E\in\mathrm{Mat}(m,n)$ such that

$$A=BE. \tag{10}$$

Proof. For any $K\in\mathrm{Mat}(m,n)$, we have that $A+BK\in\mathrm{Mat}(p,n)$ and $(A+BK)^T(A+BK)\geq 0$. Inserting $K=-(B^TB)^{-1}B^TA$ yields

$$A^TA-A^TB(B^TB)^{-1}B^TA-A^TB(B^TB)^{-1}B^TA+A^TB\underbrace{(B^TB)^{-1}B^TB(B^TB)^{-1}}_{(B^TB)^{-1}}B^TA\geq 0,$$

which proves the bound. The saturation condition $A+BK=0$ is satisfied if and only if $A=\Pi_BA$, where $\Pi_B=B(B^TB)^{-1}B^T$ is the projector onto the range of B. This is equivalent to $A=BE$ for some $E\in\mathrm{Mat}(m,n)$ and completes the proof.

For $n=m=1$ this inequality reduces to the Cauchy-Schwarz inequality for vectors. The saturation condition (10) then yields the well-known requirement that the p-dimensional vectors A and B must be parallel.

B. General properties

Convexity.—Introducing vectors A and B with matrix-valued entries $A_\gamma=\sqrt{p_\gamma}\Gamma[\hat\rho_\gamma,\hat{\mathbf{X}}]^{-\frac{1}{2}}C[\hat\rho_\gamma,\hat{\mathbf{H}},\hat{\mathbf{X}}]$ and $B_\gamma=\sqrt{p_\gamma}\Gamma[\hat\rho_\gamma,\hat{\mathbf{X}}]^{\frac{1}{2}}$, we obtain $A^TA=\sum_\gamma p_\gamma C[\hat\rho_\gamma,\hat{\mathbf{H}},\hat{\mathbf{X}}]^T\Gamma[\hat\rho_\gamma,\hat{\mathbf{X}}]^{-1}C[\hat\rho_\gamma,\hat{\mathbf{H}},\hat{\mathbf{X}}]$, $B^TB=\sum_\gamma p_\gamma\Gamma[\hat\rho_\gamma,\hat{\mathbf{X}}]$, and $B^TA=\sum_\gamma p_\gamma C[\hat\rho_\gamma,\hat{\mathbf{H}},\hat{\mathbf{X}}]$. From Supplementary Equation (9) follows that

$$\sum_\gamma p_\gamma\mathcal{M}[\hat\rho_\gamma,\hat{\mathbf{H}},\hat{\mathbf{X}}]\geq C[\hat\rho,\hat{\mathbf{H}},\hat{\mathbf{X}}]^T\left(\sum_\gamma p_\gamma\Gamma[\hat\rho_\gamma,\hat{\mathbf{X}}]\right)^{-1}C[\hat\rho,\hat{\mathbf{H}},\hat{\mathbf{X}}], \tag{11}$$

where $C[\hat\rho,\hat{\mathbf{H}},\hat{\mathbf{X}}]=\sum_\gamma p_\gamma C[\hat\rho_\gamma,\hat{\mathbf{H}},\hat{\mathbf{X}}]$ for $\hat\rho=\sum_\gamma p_\gamma\hat\rho_\gamma$. Furthermore, the concavity of the covariance matrix implies that $\left(\sum_\gamma p_\gamma\Gamma[\hat\rho_\gamma,\hat{\mathbf{X}}]\right)^{-1}\geq\Gamma[\hat\rho,\hat{\mathbf{X}}]^{-1}$ and we finally obtain the convexity property:

$$\mathcal{M}[\hat\rho,\hat{\mathbf{H}},\hat{\mathbf{X}}]\leq\sum_\gamma p_\gamma\mathcal{M}[\hat\rho_\gamma,\hat{\mathbf{H}},\hat{\mathbf{X}}]. \tag{12}$$

Orthogonal transformations.—Let us first note some general transformation properties of the covariance and commutator matrices, respectively. We first introduce a larger family of Hermitian operators $\hat{\mathbf{A}}=(\hat A_1,\dots,\hat A_L)^T$, such that we can express the elements of $\hat{\mathbf{H}}$ and $\hat{\mathbf{X}}$ as linear combinations of the elements of $\hat{\mathbf{A}}$ (expressed as a column vector):

$$\hat{\mathbf{H}}=R\hat{\mathbf{A}},\qquad\hat{\mathbf{X}}=S\hat{\mathbf{A}}. \tag{13}$$

The following property holds:

$$\Gamma[\hat\rho,\hat{\mathbf{X}}]=S\Gamma[\hat\rho,\hat{\mathbf{A}}]S^T, \tag{14}$$

where we used that $\mathrm{Cov}(\hat X_k,\hat X_l)_{\hat\rho}=\sum_{ij=1}^L s_{k,i}s_{l,j}\mathrm{Cov}(\hat A_i,\hat A_j)_{\hat\rho}$, due to the bilinearity of the covariance. Analogously,

$$C[\hat\rho,\hat{\mathbf{H}},\hat{\mathbf{X}}]=S\tilde C[\hat\rho,\hat{\mathbf{A}}]R^T \tag{15}$$

follows from the bilinearity of the commutator, i.e., $-i\langle[\hat X_k,\hat H_l]\rangle_{\hat\rho}=-i\sum_{ij=1}^L s_{k,i}r_{l,j}\langle[\hat A_i,\hat A_j]\rangle_{\hat\rho}$, and $(\tilde C[\hat\rho,\hat{\mathbf{A}}])_{ij}=-i\langle[\hat A_i,\hat A_j]\rangle_{\hat\rho}$. From the definition

$$\tilde{\mathcal{M}}[\hat\rho,\hat{\mathbf{A}}]=\tilde C[\hat\rho,\hat{\mathbf{A}}]^T\Gamma[\hat\rho,\hat{\mathbf{A}}]^{-1}\tilde C[\hat\rho,\hat{\mathbf{A}}], \tag{16}$$

and the transformation properties (14) and (15) follows for $S=R=O$, where O is an orthogonal matrix, that

$$\tilde{\mathcal{M}}[\hat\rho,O\hat{\mathbf{A}}]=O\tilde{\mathcal{M}}[\hat\rho,\hat{\mathbf{A}}]O^T. \tag{17}$$

The matrix O can be chosen to diagonalize the moment matrix.

C. Maximizing the moment matrix

Here we maximize the moment matrix (8) over the measurement observables $\hat{\mathbf{X}}$ as a function of a family of measurable operators $\hat{\mathbf{A}} = (\hat{A}_1, \dots, \hat{A}_L)^T$. Let $\hat{\mathbf{X}} = (\hat{X}_1, \dots, \hat{X}_K)^T$ and $\hat{\mathbf{H}} = (\hat{H}_1, \dots, \hat{H}_M)^T$, i.e., $R \in \mathrm{Mat}(M, L)$ and $S \in \mathrm{Mat}(K, L)$.

Inserting Supplementary Equations (14) and (15) into Supplementary Equation (8) yields the following expression for the moment matrix:

$$\mathcal{M}[\hat{\rho}, \hat{\mathbf{H}}, \hat{\mathbf{X}}] = R\tilde{C}[\hat{\rho}, \hat{\mathbf{A}}]^T S^T \left(S\Gamma[\hat{\rho}, \hat{\mathbf{A}}]S^T\right)^{-1} S\tilde{C}[\hat{\rho}, \hat{\mathbf{A}}]R^T.$$

We now apply the inequality (9) with $A = \Gamma[\hat{\rho}, \hat{\mathbf{A}}]^{-\frac{1}{2}}\tilde{C}[\hat{\rho}, \hat{\mathbf{A}}]R^T$ and $B = \Gamma[\hat{\rho}, \hat{\mathbf{A}}]^{\frac{1}{2}}S^T$, leading to

$$\mathcal{M}[\hat{\rho}, \hat{\mathbf{H}}, \hat{\mathbf{X}}] \leq R\tilde{\mathcal{M}}[\hat{\rho}, \hat{\mathbf{A}}]R^T, \tag{18}$$

with $\tilde{\mathcal{M}}[\hat{\rho}, \hat{\mathbf{A}}]$ defined in Supplementary Equation (16).

Inequality (18) provides an upper bound on the moment-based multiparameter sensitivity for any choice of the observables $\hat{\mathbf{X}}$. The maximal sensitivity is reached when the inequality is saturated. The saturation condition (10) is fulfilled when

$$GS = R\tilde{C}[\hat{\rho}, \hat{\mathbf{A}}]^T\Gamma[\hat{\rho}, \hat{\mathbf{A}}]^{-1}, \tag{19}$$

for some $G \in \mathrm{Mat}(M, K)$, which corresponds to $G = E^T$ in Supplementary Equation (10). Recall that the matrix S determines the measurement operators $\hat{\mathbf{X}}$ via Supplementary Equation (13). The freedom provided by the matrix G can be used to rearrange and normalize the measurement observables. When we have as many measurement observables as there are parameters $K = M$, we can choose $G = T^{-1}$, leading to the expression

$$\hat{\mathbf{X}}_{\mathrm{opt}} = TR\tilde{C}[\hat{\rho}, \hat{\mathbf{A}}]^T\Gamma[\hat{\rho}, \hat{\mathbf{A}}]^{-1}\hat{\mathbf{A}}, \tag{20}$$

where T is an arbitrary invertible $M \times M$ matrix. By demonstrating the saturability of the lower bound (18), we have solved the maximization problem of the moment matrix over all measurement operators $\hat{\mathbf{X}}$ from the accessible set $\hat{\mathbf{A}}$ for fixed $\hat{\mathbf{H}}$. In practice, saturation can be achieved when all elements of $\hat{\mathbf{X}}_{\mathrm{opt}}$ can be measured simultaneously.

D. Lower bound on the classical Fisher matrix

Here, we demonstrate a general result that implies

$$\max_{\hat{\mathbf{X}} \in \mathrm{span}(\hat{\mathbf{\Pi}})} \mathcal{M}[\hat{\rho}(\theta), \hat{\mathbf{H}}, \hat{\mathbf{X}}] = F[\hat{\rho}(\theta), \hat{\mathbf{X}}] \tag{21}$$

for the special case of unitary evolution. We denote the spectral decomposition of the observables as $\hat{X}_l = \sum_k x_l(k)\hat{\Pi}_k$, where the $\hat{\Pi}_k$ are the projectors onto a common eigenbasis of all $\hat{X}_l$. The quantum mechanical expectation values are given by $\langle\hat{X}_l\rangle_{\hat{\rho}(\theta)} = \sum_k x_l(k)p(k|\theta)$ with $p(k|\theta) = \mathrm{Tr}\{\hat{\rho}(\theta)\hat{\Pi}_k\}$.

Let us now consider the matrix bound (9) with matrices $A_{kl} = \sqrt{p(k|\theta)}\left(\frac{\partial}{\partial\theta_l}\log p(k|\theta)\right)$ and $B_{kl} = \sqrt{p(k|\theta)}\left(x_l(k) - \langle\hat{X}_l\rangle_{\hat{\rho}(\theta)}\right)$. We obtain

$$\begin{aligned} A^TA &= F[\hat{\rho}(\theta), \hat{\mathbf{X}}], \\ B^TB &= \Gamma[\hat{\rho}(\theta), \hat{\mathbf{X}}], \\ B^TA &= D[\hat{\rho}(\theta), \hat{\mathbf{X}}], \end{aligned} \tag{22}$$

and (9) implies that

$$\mathcal{M}[\hat{\rho}(\theta), \hat{\mathbf{H}}, \hat{\mathbf{X}}] \leq F[\hat{\rho}(\theta), \hat{\mathbf{X}}], \tag{23}$$

for any $\hat{\rho}(\theta)$ and any $\hat{\mathbf{X}}$.

Saturation condition.—Let us now discuss the conditions for the saturation of (23). A straightforward solution to the saturation condition (10) is obtained by requiring that $A = B$, which is achieved by the choice

$$x_l(k) = \frac{\partial}{\partial\theta_l}\log p(k|\theta) + \langle\hat{X}_l\rangle_{\hat{\rho}(\theta)}, \tag{24}$$

for all k and l. This means that a measurement of the observables

$$\hat{X}_l = \sum_k \left(\frac{\partial}{\partial \theta_l} \log p(k|\theta) + \langle \hat{X}_l \rangle_{\hat{\rho}(\theta)} \right) \hat{\Pi}_k. \tag{25}$$

leads to saturation of the bound (23), i.e., the moment matrix associated with these observables coincides with the Fisher matrix generated by the projectors in their common eigenbasis. Notice that transformations of the type $\hat{X}_l \to \alpha \hat{X}_l + \beta \hat{\mathbb{I}}_l$ with arbitrary $\alpha, \beta \in \mathbb{R}$ do not alter the moment matrix.

Saturating the bound by measuring projectors.—Alternatively, we may consider the special case where the projectors $\hat{\Pi}_k$ themselves are the measurement observables and we estimate the parameters θ from the average values of $\hat{\mathbf{X}} = \hat{\mathbf{\Pi}}$. Recall that the moment matrix (6) depends on the inverse of the covariance matrix of the measured observables. Singularities of the covariance matrix may arise either due to redundant information from the measurement of too many projectors that span a complete basis, or from projectors that are orthogonal to the state and lead to $p(k|\theta) = \langle \hat{\Pi}_k \rangle_{\rho(\theta)} = 0$. This can be avoided by effectively limiting the set of measured observables $\hat{\mathbf{X}} = \{\hat{\Pi}_k\}_{k=1}^{d-1}$ to a subset of $d-1$ projectors such that $\sum_{k=1}^{d-1} p(k|\theta) = 1 - p(d|\theta) < 1$. We obtain from Supplementary Equation (7) that $D[\hat{\rho}(\theta), \hat{\mathbf{X}}]_{kl} = \frac{\partial p(k|\theta)}{\partial \theta_l}$ and [5]

$$\Gamma[\hat{\rho}(\theta), \hat{\mathbf{X}}] = P(\theta) - \mathbf{p}(\theta)\mathbf{p}(\theta)^T, \tag{26}$$

where $P(\theta) = \mathrm{diag}(p(1|\theta), \dots, p(d-1|\theta))$ and $\mathbf{p}(\theta) = (p(1|\theta), \dots, p(d-1|\theta))^T$. Using $\Gamma[\hat{\rho}(\theta), \hat{\mathbf{X}}]^{-1} = P(\theta)^{-1} + \frac{1}{p(d|\theta)} \mathbf{e}\mathbf{e}^T$ with $\mathbf{e} = (1, \dots, 1)^T$ we obtain [for unitary evolutions, this coincides with $\mathcal{M}[\hat{\rho}(\theta), \hat{\mathbf{H}}, \hat{\mathbf{X}}]$ defined in Supplementary Equation (8)]

$$\mathcal{M}[\hat{\rho}(\theta), \hat{\mathbf{X}}] = D[\hat{\rho}(\theta), \hat{\mathbf{X}}]^T P(\theta)^{-1} D[\hat{\rho}(\theta), \hat{\mathbf{X}}] + \frac{1}{p(d|\theta)} \left(D[\hat{\rho}(\theta), \hat{\mathbf{X}}]^T \mathbf{e} \right) \left(D[\hat{\rho}(\theta), \hat{\mathbf{X}}]^T \mathbf{e} \right)^T. \tag{27}$$

Using

$$\left(D[\hat{\rho}(\theta), \hat{\mathbf{X}}]^T P(\theta)^{-1} D[\hat{\rho}(\theta), \hat{\mathbf{X}} \right)_{kl} = \sum_{m=1}^{d-1} \frac{1}{p(m|\theta)} \left(\frac{\partial p(m|\theta)}{\partial \theta_k} \right) \left(\frac{\partial p(m|\theta)}{\partial \theta_l} \right) \tag{28}$$

and

$$\left(D[\hat{\rho}(\theta), \hat{\mathbf{X}}]^T \mathbf{e} \right)_k = \sum_{l=1}^{d-1} \frac{\partial p(l|\theta)}{\partial \theta_k} = -\frac{\partial p(d|\theta)}{\partial \theta_k}, \tag{29}$$

we obtain

$$\mathcal{M}[\hat{\rho}(\theta), \hat{\mathbf{X}}]_{kl} = \sum_{m=1}^{d} p(m|\theta) \left(\frac{\partial}{\partial \theta_k} \log p(m|\theta) \right) \left(\frac{\partial}{\partial \theta_l} \log p(m|\theta) \right), \tag{30}$$

which is the Fisher matrix $F[\hat{\rho}(\theta), \hat{\mathbf{X}}]$.

We end this section with two remarks: First, we note that we obtain a sensitivity of the form (30) even if the projectors are not of rank one, i.e., when we coarse-grain over measurement outcomes. Second, the result holds for arbitrary choices of the projectors even in the case of non-commuting generators. For pure probe states, necessary and sufficient conditions given in Supplementary Reference [6] reveal whether a chosen set of projectors also saturates the quantum Fisher matrix.

E. Lower bound on the quantum Fisher matrix

Here, we generalize and demonstrate

$$\mathcal{M}[\hat{\rho}, \hat{\mathbf{H}}, \hat{\mathbf{X}}] \leq F_Q[\hat{\rho}, \hat{\mathbf{H}}]. \tag{31}$$

Let us first demonstrate that the lower bound

$$\tilde{\mathcal{M}}[\hat{\rho}, \hat{\mathbf{A}}] \leq F_Q[\hat{\rho}, \hat{\mathbf{A}}], \tag{32}$$

holds for arbitrary families of operators $\hat{\mathbf{A}}$. Here, $(F_Q[\hat{\rho}, \hat{\mathbf{A}}])_{ij} = \mathrm{Tr}\{\hat{\rho}(\hat{\mathcal{L}}_i \hat{\mathcal{L}}_j + \hat{\mathcal{L}}_j \hat{\mathcal{L}}_i)/2\}$ are the elements of the quantum Fisher matrix with symmetric logarithmic derivatives defined as the solution to $-i[\hat{A}_j, \hat{\rho}] = (\hat{\mathcal{L}}_j \hat{\rho} + \hat{\rho} \hat{\mathcal{L}}_j)/2$ [7].

To prove inequality (32), note that for an arbitrary $\mathbf{n} = (n_1, \dots, n_L)^T \in \mathbb{R}^L$, the equality $\mathbf{n}^T \tilde{\mathcal{M}}[\hat{\rho}, \hat{\mathbf{A}}]\mathbf{n} = |\langle[\hat{H}_{\mathbf{m}_{\rm opt}}, \hat{H}_{\mathbf{n}}]\rangle_{\hat{\rho}}|^2/(\Delta\hat{H}_{\mathbf{m}_{\rm opt}})^2_{\hat{\rho}}$ is achieved for an optimally chosen $\hat{H}_{\mathbf{m}} = \mathbf{m}^T\hat{\mathbf{A}}$, where $\mathbf{m}_{\rm opt} = \alpha\Gamma[\hat{\rho}, \hat{\mathbf{A}}]^{-1}\tilde{C}[\hat{\rho}, \hat{\mathbf{A}}]\mathbf{n}$ with some normalization constant α [8]. Furthermore, the inequality $|\langle[\hat{H}_{\mathbf{m}}, \hat{H}_{\mathbf{n}}]\rangle_{\hat{\rho}}|^2/(\Delta\hat{H}_{\mathbf{m}})^2_{\hat{\rho}} \leq \mathbf{n}^T F_Q[\hat{\rho}, \hat{\mathbf{A}}]\mathbf{n}$ holds for all $\mathbf{n}, \mathbf{m} \in \mathbb{R}^L$ and saturation is achieved for $\hat{H}_{\mathbf{m}} = \hat{L}_{\mathbf{n}}$ [8], where $\hat{L}_{\mathbf{n}}$ is defined as the solution to the equation

$$-i[\hat{H}_{\mathbf{n}}, \hat{\rho}] = (\hat{L}_{\mathbf{n}}\hat{\rho} + \hat{\rho}\hat{L}_{\mathbf{n}})/2. \tag{33}$$

We thus obtain $\mathbf{n}^T\tilde{\mathcal{M}}[\hat{\rho}, \hat{\mathbf{A}}]\mathbf{n} \leq \mathbf{n}^T F_Q[\hat{\rho}, \hat{\mathbf{A}}]\mathbf{n}$ for all $\mathbf{n}$, demonstrating the statement (32).

Let us now turn our attention to the saturation condition. The above derivation shows that the equality $\tilde{\mathcal{M}}[\hat{\rho}, \hat{\mathbf{A}}] = F_Q[\hat{\rho}, \hat{\mathbf{A}}]$ can be achieved if for all $\mathbf{n}$, the $\hat{L}_{\mathbf{n}}$ can be expressed as linear combinations of the elements of $\hat{\mathbf{A}}$. Using the linearity of the condition (33), we find that $L_{\mathbf{n}} = \mathbf{n}^T\hat{\mathcal{L}}$, where $\hat{\mathcal{L}} = (\hat{\mathcal{L}}_1, \dots, \hat{\mathcal{L}}_L)^T$. A sufficient saturation condition is therefore that for each $\hat{A}_j$, also the corresponding $\hat{\mathcal{L}}_j$ is an element of $\hat{\mathbf{A}}$.

Equation (31) now follows from (32) by using $F_Q[\hat{\rho}, \hat{\mathbf{H}}] = RF_Q[\hat{\rho}, \hat{\mathbf{A}}]R^T$ [9] and Supplementary Equation (18). The maximum can be attained if the optimal set of observables (19) can be measured simultaneously.

III. SUPPLEMENTARY NOTE 3: SPIN SQUEEZING MATRIX

This section discusses properties of the squeezing matrix in the case of discrete variables. Linear parameter-encoding Hamiltonians $\hat{\mathbf{H}}$ and measurement observables $\hat{\mathbf{X}}$ of a collective spin system can be expressed in terms of the $3M$ angular momentum operators $\hat{\mathbf{J}} = (\hat{\mathbf{J}}_1^T, ..., \hat{\mathbf{J}}_M^T)^T$ with $\hat{\mathbf{J}}_k = (\hat{J}_{x,k}, \hat{J}_{y,k}, \hat{J}_{z,k})^T$.

A. Accessible operators for collective spin systems

Let us first consider the full vector $\hat{\mathbf{J}}$ as family of accessible operators. In this case we obtain the commutator matrix

$$\tilde{C}[\hat{\rho}, \hat{\mathbf{J}}] = \bigoplus_{k=1}^{M} \begin{pmatrix} 0 & \langle\hat{J}_{z,k}\rangle_{\hat{\rho}} & -\langle\hat{J}_{y,k}\rangle_{\hat{\rho}} \\ -\langle\hat{J}_{z,k}\rangle_{\hat{\rho}} & 0 & \langle\hat{J}_{x,k}\rangle_{\hat{\rho}} \\ \langle\hat{J}_{y,k}\rangle_{\hat{\rho}} & -\langle\hat{J}_{x,k}\rangle_{\hat{\rho}} & 0 \end{pmatrix}. \tag{34}$$

As a skew-symmetric matrix with odd dimension $3M$, the matrix (34) is singular due to Jacobi's theorem. The singularity can be avoided by restricting the operator basis to the relevant subset. We define a local mean spin direction $\mathbf{n}_{0,k} = \langle\hat{\mathbf{J}}_k\rangle_{\hat{\rho}}/|\langle\hat{\mathbf{J}}_k\rangle_{\hat{\rho}}|$, which can be extended by two orthogonal vectors $\mathbf{n}_{\perp_1,k}$ and $\mathbf{n}_{\perp_2,k}$ to a complete basis. We can choose any three orthogonal spin operators as a basis to represent linear operators $\hat{H}_k$ and $\hat{X}_k$. Locally rearranging the basis to align the mean field direction $\mathbf{n}_{0,k}$ onto the z-direction leads to the commutator matrix

$$\tilde{C}[\hat{\rho}, \hat{\mathbf{J}}] = \bigoplus_{k=1}^{M} \begin{pmatrix} 0 & \langle\hat{J}_{z,k}\rangle_{\hat{\rho}} & 0 \\ -\langle\hat{J}_{z,k}\rangle_{\hat{\rho}} & 0 & 0 \\ 0 & 0 & 0 \end{pmatrix}. \tag{35}$$

As a consequence, the rows and columns of $\tilde{\mathcal{M}}[\hat{\rho}, \hat{\mathbf{J}}]$ [see Supplementary Equation (16)] belonging to $\hat{J}_{z,k}$ remain zero and it suffices to restrict to the two-dimensional subspace spanned by $\mathbf{n}_{\perp_1,k}$ and $\mathbf{n}_{\perp_2,k}$. In this subspace, the matrix $\tilde{C}[\hat{\rho}, \hat{\mathbf{J}}_\perp] = \bigoplus_{k=1}^{M} |\langle\hat{\mathbf{J}}_k\rangle_{\hat{\rho}}|\omega$ with $\omega = \left(\begin{smallmatrix} 0 & 1 \\ -1 & 0 \end{smallmatrix}\right)$ is always invertible and reflects the symplectic form of canonical transformations, that prominently appears in the description of continuous-variable systems [10–12], locally rescaled by the spin length $\langle\hat{J}_{z,k}\rangle_{\hat{\rho}} = |\langle\hat{\mathbf{J}}_k\rangle_{\hat{\rho}}|$. For simplicity, we henceforth choose a basis described by $\{\mathbf{e}_x, \mathbf{e}_y, \mathbf{e}_z\} = \{\mathbf{n}_{\perp_1,k}, \mathbf{n}_{\perp_2,k}, \mathbf{n}_{0,k}\}$, such that $\hat{\mathbf{J}}_\perp = (\hat{J}_{x,1}, \hat{J}_{y,1}, \dots, \hat{J}_{x,M}, \hat{J}_{y,M})^T$. The operator basis $\hat{\mathbf{J}}_\perp$ is sufficient for our purposes since measurements and evolutions that involve the mean spin direction $\mathbf{n}_{0,k}$ are suboptimal for metrology.

B. Multiparameter shot-noise limit

Here we identify the shot-noise limit for the quantum Fisher matrix generated by the set of accessible operators $\hat{\mathbf{J}}_\perp$. First, we consider the larger set $\hat{\mathbf{J}}$ and we obtain $\sup_{\hat{\rho}_{\rm p-sep}} F_Q[\hat{\rho}_{\rm p-sep}, \hat{\mathbf{J}}] = \sup_{|\Psi^{(1)}\rangle\otimes\cdots\otimes|\Psi^{(N)}\rangle} 4\Gamma[|\Psi^{(1)}\rangle \otimes \cdots \otimes |\Psi^{(N)}\rangle, \hat{\mathbf{J}}]$ due to the convexity of the quantum Fisher matrix [9]. Let us now express the vector $\hat{\mathbf{J}}$ as a sum of single-particle vectors $\hat{\mathbf{J}} = \sum_{i=1}^{N}\hat{\mathbf{J}}^{(i)}$, where $\hat{\mathbf{J}}^{(i)} = (\hat{j}^{(i)}_{x,1}, \hat{j}^{(i)}_{y,1}, \hat{j}^{(i)}_{z,1}, \hat{j}^{(i)}_{x,2}, \hat{j}^{(i)}_{y,2}, \hat{j}^{(i)}_{z,2}, \dots, \hat{j}^{(i)}_{x,M}, \hat{j}^{(i)}_{y,M}, \hat{j}^{(i)}_{z,M})^T$. The operators $\hat{j}^{(i)}_{\alpha,k} = \frac{1}{2}\hat{\Pi}^{(i)}_k\hat{\sigma}^{(i)}_{\alpha,k}\hat{\Pi}^{(i)}_k$ contain the projectors $\hat{\Pi}^{(i)}_k$ of particle i

onto the subspace of mode k, such that $\hat{\Pi}_k^{(i)}\hat{\Pi}_l^{(j)} = \delta_{kl}\delta_{ij}\hat{\Pi}_k^{(i)}$. We may expand the single-particle states as $|\Psi^{(i)}\rangle = \sum_{k=1}^{M}\sqrt{p_k^{(i)}}|\Psi_k^{(i)}\rangle$, with orthonormal local states $\hat{\Pi}_l^{(i)}|\Psi_k^{(i)}\rangle = \delta_{kl}|\Psi_k^{(i)}\rangle$ and $p_k^{(i)}$ denotes the probability for particle i to be in mode k with $\sum_{k=1}^{M} p_k^{(i)} = 1$. Using $\mathit{\Gamma}[|\Psi^{(1)}\rangle\otimes\cdots\otimes|\Psi^{(N)}\rangle, \hat{\mathbf{J}}] = \sum_{i=1}^{N}\mathit{\Gamma}[|\Psi^{(i)}\rangle, \hat{\mathbf{J}}^{(i)}]$ and discarding the first moments [9], we obtain

$$\mathit{\Gamma}[|\Psi^{(1)}\rangle\otimes\cdots\otimes|\Psi^{(N)}\rangle, \hat{\mathbf{J}}] \leq \sum_{i=1}^{N}\begin{pmatrix} p_1^{(i)}\tilde{\mathit{\Gamma}}[|\Psi_1^{(i)}\rangle, \hat{\mathbf{j}}_1^{(i)}] & \mathit{0} & \cdots & \mathit{0} \\ \vdots & & \ddots & \vdots \\ \mathit{0} & & \cdots\ \mathit{0} & p_M^{(i)}\tilde{\mathit{\Gamma}}[|\Psi_M^{(i)}\rangle, \hat{\mathbf{j}}_M^{(i)}] \end{pmatrix}, \tag{36}$$

where $\hat{\mathbf{j}}_k^{(i)} = (\hat{j}_{x,k}^{(i)}, \hat{j}_{y,k}^{(i)}, \hat{j}_{z,k}^{(i)})^T$, the $\tilde{\mathit{\Gamma}}[|\Psi_k^{(i)}\rangle, \hat{\mathbf{j}}_k^{(i)}]$ are 3×3 matrices with elements $(\tilde{\mathit{\Gamma}}[|\Psi_k^{(i)}\rangle, \hat{\mathbf{j}}_k^{(i)}])_{\alpha\beta} = \frac{1}{2}\langle \hat{j}_{\alpha,k}^{(i)}\hat{j}_{\beta,k}^{(i)} + \hat{j}_{\beta,k}^{(i)}\hat{j}_{\alpha,k}^{(i)}\rangle_{|\Psi_k^{(i)}\rangle} = \frac{1}{8}\langle \hat{\sigma}_{\alpha,k}^{(i)}\hat{\sigma}_{\beta,k}^{(i)} + \hat{\sigma}_{\beta,k}^{(i)}\hat{\sigma}_{\alpha,k}^{(i)}\rangle_{|\Psi_k^{(i)}\rangle}$, and $\mathit{0}$ is the 3×3 zero matrix. Using the anticommutativity property of the Pauli matrices we obtain that $\tilde{\mathit{\Gamma}}[|\Psi_k^{(i)}\rangle, \hat{\mathbf{j}}_k^{(i)}] = \frac{1}{4}\mathit{1}$ for all k, i and arbitrary $|\Psi_k^{(i)}\rangle$, where $\mathit{1}$ is the 3×3 identity matrix. The upper bound $F_Q[\hat{\rho}_{\text{p-sep}}, \hat{\mathbf{J}}] \leq \operatorname{diag}(N_1\mathit{1}, \ldots, N_M\mathit{1})$ is obtained by inserting this back into Supplementary Equation (36), and using that $\sum_{i=1}^{N} p_k^{(i)} = N_k$ is the average number of particles in mode k. This upper bound cannot be saturated since not all first moments can be zero simultaneously for a pure single-qubit state. However, by restricting the set of accessible operators to the two directions orthogonal to the mean-spin direction $\mathbf{n}_{0,k}$, we obtain an analogous result with 2×2 instead of 3×3 blocks, i.e., $F_Q[\hat{\rho}_{\text{p-sep}}, \hat{\mathbf{J}}_\perp] \leq \operatorname{diag}(N_1, N_1, \ldots, N_M, N_M)$. This bound is saturated by single-qubit states $|\Psi_k^{(i)}\rangle$ that are polarized along $\mathbf{n}_{0,k}$.

C. Local squeezing

In the case of an uncorrelated product of single-mode squeezed states $\hat{\rho}_{\text{loc}} = \hat{\rho}_1\otimes\cdots\otimes\hat{\rho}_M$, the moment matrix attains a block-diagonal form, $\tilde{\mathcal{M}}[\hat{\rho}_{\text{loc}}, \hat{\mathbf{J}}_\perp] = \bigoplus_{k=1}^{M}\tilde{\mathcal{M}}[\hat{\rho}_k, \hat{\mathbf{J}}_{\perp,k}]$. Furthermore, $F_{\text{SN}}[\hat{\mathbf{H}}] = RF_{\text{SN}}[\hat{\mathbf{J}}_\perp]R^T = \operatorname{diag}(N_1, \ldots, N_M)$ implies that the squeezing matrix, optimized over all measurement observables, reads

$$\begin{aligned} \mathit{\Xi}^2_{\text{opt}}[\hat{\rho}_{\text{loc}}, \hat{\mathbf{H}}, \hat{\mathbf{J}}_\perp] &= \min_{\hat{\mathbf{X}}\in\operatorname{span}(\hat{\mathbf{A}})}\mathit{\Xi}^2[\hat{\rho}_{\text{loc}}, \hat{\mathbf{H}}, \hat{\mathbf{X}}] \\ &= F_{\text{SN}}[\hat{\mathbf{H}}]^{\frac{1}{2}}R\tilde{\mathcal{M}}[\hat{\rho}_{\text{loc}}, \hat{\mathbf{J}}_\perp]^{-1}R^TF_{\text{SN}}[\hat{\mathbf{H}}]^{\frac{1}{2}} \\ &= R\bigoplus_{k=1}^{M}N_k\tilde{\mathcal{M}}[\hat{\rho}_k, \hat{\mathbf{J}}_{\perp,k}]^{-1}R^T. \end{aligned} \tag{37}$$

1. *Optimizing the phase-imprinting Hamiltonians*

An optimal choice for R is provided when the eigenvalues of the $M\times M$ matrix $\mathit{\Xi}^2_{\text{opt}}$ correspond to the M smallest eigenvalues of the $2M\times 2M$ matrix $\bigoplus_{k=1}^{M}N_k\tilde{\mathcal{M}}[\hat{\rho}_k, \hat{\mathbf{J}}_{\perp,k}]^{-1}$ (see also the discussion in the Methods section). Let us therefore consider the eigenvalues of this matrix. Each of the 2×2 blocks can be written as

$$N_k\tilde{\mathcal{M}}[\hat{\rho}_k, \hat{\mathbf{J}}_{\perp,k}]^{-1} = \frac{N_k}{\langle\hat{J}_{z,k}\rangle^2_{\hat{\rho}_k}}\omega\mathit{\Gamma}[\hat{\rho}_k, \hat{\mathbf{J}}_{\perp,k}]\omega^T, \tag{38}$$

where we used that $C[\hat{\rho}_k, \hat{\mathbf{J}}_{\perp,k}] = \langle\hat{J}_{z,k}\rangle_{\hat{\rho}_k}\omega$ with $\omega = \begin{pmatrix} 0 & 1 \\ -1 & 0\end{pmatrix}$ [see Supplementary Equation (35)]. The eigenvalues

$$\begin{aligned} \lambda_{-,k} &= N_k\min_{\substack{\mathbf{r}_k \\ |\mathbf{r}_k|^2=1}}\mathbf{r}_k^T\tilde{\mathcal{M}}[\hat{\rho}_k, \hat{\mathbf{J}}_{\perp,k}]^{-1}\mathbf{r}_k = N_k\lambda_{\max}(\tilde{\mathcal{M}}[\hat{\rho}_k, \hat{\mathbf{J}}_{\perp,k}])^{-1}, \\ \lambda_{+,k} &= N_k\max_{\substack{\mathbf{r}_k \\ |\mathbf{r}_k|^2=1}}\mathbf{r}_k^T\tilde{\mathcal{M}}[\hat{\rho}_k, \hat{\mathbf{J}}_{\perp,k}]^{-1}\mathbf{r}_k = N_k\lambda_{\min}(\tilde{\mathcal{M}}[\hat{\rho}_k, \hat{\mathbf{J}}_{\perp,k}])^{-1} \end{aligned}$$

correspond to a squeezed and an anti-squeezed variance (renormalized by the mean spin length), respectively. Indeed, $\lambda_{-,k}$ can be identified as the single-mode spin-squeezing coefficient of mode k [13–16], optimized over all local measurements and evolutions [8], $\lambda_{-,k} = \xi^2_{\min}[\hat{\rho}_k, \hat{\mathbf{J}}_{\perp,k}] = N_k\min_{\mathbf{r}_k,\mathbf{s}_k}|\langle[\hat{J}_{\mathbf{s}_k,k}, \hat{J}_{\mathbf{r}_k,k}]\rangle_{\hat{\rho}_k}|^{-2}(\Delta\hat{J}_{\mathbf{s}_k})^2_{\hat{\rho}_k}$. The uncertainty relation $(\Delta\hat{J}_{x,k})_{\hat{\rho}_k}(\Delta\hat{J}_{y,k})_{\hat{\rho}_k} \geq |\langle\hat{J}_{z,k}\rangle_{\hat{\rho}_k}|/2$ excludes that both directions of the same mode can be simultaneously squeezed, i.e., $\xi^2_{\min}[\hat{\rho}_k, \hat{\mathbf{J}}_{\perp,k}] < 1$ implies that $\lambda_{+,k} > 1$: We have $\operatorname{Tr}\{\mathit{\Gamma}[\hat{\rho}_k, \hat{\mathbf{J}}_{\perp,k}]\} = (\Delta\hat{J}_{x,k})^2_{\hat{\rho}_k} + (\Delta\hat{J}_{y,k})^2_{\hat{\rho}_k} \geq 2(\Delta\hat{J}_{x,k})_{\hat{\rho}_k}(\Delta\hat{J}_{y,k})_{\hat{\rho}_k} \geq |\langle\hat{J}_{z,k}\rangle_{\hat{\rho}_k}|$, which leads to $\lambda_{+,k} + \lambda_{-,k} = N_k\operatorname{Tr}\{\tilde{\mathcal{M}}[\hat{\rho}_k, \hat{\mathbf{J}}_{\perp,k}]^{-1}\} =$

$N_k\langle\hat{J}_{z,k}\rangle^{-2}\mathrm{Tr}\{\Gamma[\hat{\rho}_k,\hat{\mathbf{J}}_{\perp,k}]\} \geq N_k/|\langle\hat{J}_{z,k}\rangle_{\hat{\rho}_k}|$. Hence, $\xi^2_{\min}[\hat{\rho}_k,\hat{\mathbf{J}}_{\perp,k}] = \lambda_{-,k} < 1$ implies that $1 > \lambda_{-,k} \geq N_k/|\langle\hat{J}_{z,k}\rangle_{\hat{\rho}_k}| - \lambda_{+,k} \geq 2 - \lambda_{+,k}$, whence $\lambda_{+,k} > 1$, and we used that $|\langle\hat{J}_{z,k}\rangle_{\hat{\rho}_k}| \leq N_k/2$.

Assuming that local squeezing is present in each mode, we conclude that it is optimal to encode all M parameters into the respective squeezed local variables that correspond to the eigenvalues $\lambda_{-,k}$ for $k = 1,\ldots,M$. Formally this is achieved by a transformation matrix of the form

$$R = \begin{pmatrix} r_{x,1} & r_{y,1} & 0 & 0 & \cdots & 0 \\ 0 & 0 & r_{x,2} & r_{y,2} & \cdots & 0 \\ \vdots & & & \ddots & & \vdots \\ 0 & 0 & & \cdots & r_{x,M} & r_{y,M} \end{pmatrix}, \tag{39}$$

where $\mathbf{r}_k = (r_{x,k}, r_{y,k})^T$ is normalized. Minimizing over the local directions $\mathbf{r}_k$ yields with $\hat{\mathbf{H}} = R\hat{\mathbf{J}}_\perp = \hat{\mathbf{J}}_\mathbf{r}$:

$$\begin{aligned}\xi^2_{\min}[\hat{\rho}_{\mathrm{loc}},\hat{\mathbf{J}}_\perp] &:= \min_{\mathbf{r}_1,\ldots,\mathbf{r}_M} \Xi^2_{\mathrm{opt}}[\hat{\rho}_{\mathrm{loc}},\hat{\mathbf{J}}_\mathbf{r},\hat{\mathbf{J}}_\perp] \\ &= \min_{\mathbf{r}_1,\ldots,\mathbf{r}_M} \bigoplus_{k=1}^M N_k\mathbf{r}_k^T\tilde{\mathcal{M}}[\hat{\rho}_k,\hat{\mathbf{J}}_{\perp,k}]^{-1}\mathbf{r}_k \\ &= \bigoplus_{k=1}^M \xi^2_{\min}[\hat{\rho}_k,\hat{\mathbf{J}}_{\perp,k}].\end{aligned} \tag{40}$$

Using Supplementary Equation (38), we further find

$$\xi^2_{\min}[\hat{\rho}_k,\hat{\mathbf{J}}_{\perp,k}] = \frac{N_k}{\langle\hat{J}_{z,k}\rangle^2_{\hat{\rho}_k}}\left(\Delta_+ - \sqrt{\mathrm{Cov}(\hat{J}_{x,k},\hat{J}_{y,k})^2_{\hat{\rho}_k} + \Delta_-^2}\right) \tag{41}$$

with $2\Delta_\pm = (\Delta\hat{J}_{x,k})^2_{\hat{\rho}_k} \pm (\Delta\hat{J}_{y,k})^2_{\hat{\rho}_k}$.

Inserting Supplementary Equation (39) into (20), it follows immediately that the block-diagonal structures of the matrices $\Gamma[\hat{\rho}_{\mathrm{loc}},\hat{\mathbf{J}}_\perp]$ and $\tilde{C}[\hat{\rho}_{\mathrm{loc}},\hat{\mathbf{J}}_\perp]$ (which is block-diagonal for all states) allow for a local set of optimal measurement observables. It follows that local parameter encodings and local measurements are indeed optimal for products of locally squeezed states.

We also remark that we may use the form (34) to show explicitly that for local evolutions $\hat{H}_{\mathbf{r}_k,k}$ and measurements $\hat{X}_{\mathbf{s}_k,k}$, it is favorable to choose the vectors $\mathbf{r}_k$ and $\mathbf{s}_k$ orthogonal to each other and to the mean spin direction $\mathbf{n}_{0,k}$. To see this, note that mode-local $M\times 3M$ transformation matrices $R = \mathrm{diag}(\mathbf{r}_1^T,\ldots,\mathbf{r}_M^T)$ and $S = \mathrm{diag}(\mathbf{s}_1^T,\ldots,\mathbf{s}_M^T)$ lead to $S\tilde{C}[\hat{\rho},\hat{\mathbf{J}}]R^T = \mathrm{diag}(\mathbf{s}_1^T(\mathbf{r}_1\times\langle\hat{\mathbf{J}}_1\rangle_{\hat{\rho}}),\ldots,\mathbf{s}_M^T(\mathbf{r}_M\times\langle\hat{\mathbf{J}}_M\rangle_{\hat{\rho}}))$. The diagonal elements are therefore proportional to the volume spanned by the unit vectors $\mathbf{r}_k$, $\mathbf{s}_k$ and $\mathbf{n}_{0,k}$, which is maximized by an orthogonal configuration.

2. *Local spin squeezing matrix*

In summary, in absence of mode correlations, a set of local measurement observables $\hat{\mathbf{J}}_\mathbf{s} = (\hat{J}_{\mathbf{s}_1,1},\ldots,\hat{J}_{\mathbf{s}_M,M})^T$ and phase-imprinting Hamiltonians $\hat{\mathbf{J}}_\mathbf{r} = (\hat{J}_{\mathbf{r}_1,1},\ldots,\hat{J}_{\mathbf{r}_M,M})^T$ is optimal. For any choice of $\hat{\mathbf{J}}_\mathbf{r}$ and $\hat{\mathbf{J}}_\mathbf{s}$, we obtain a diagonal squeezing matrix

$$\Xi^2[\hat{\rho}_{\mathrm{loc}},\hat{\mathbf{J}}_\mathbf{r},\hat{\mathbf{J}}_\mathbf{s}] = \begin{pmatrix} \xi^2[\hat{\rho}_1,\hat{J}_{\mathbf{r}_1,1},\hat{J}_{\mathbf{s}_1,1}] & \cdots & 0 \\ \vdots & \ddots & \vdots \\ 0 & \cdots & \xi^2[\hat{\rho}_M,\hat{J}_{\mathbf{r}_M,M},\hat{J}_{\mathbf{s}_M,M}] \end{pmatrix}, \tag{42}$$

and an additional optimization of each of the $\xi^2[\hat{\rho}_k,\hat{J}_{\mathbf{r}_k,k},\hat{J}_{\mathbf{s}_k,k}] = N_k(\Delta\hat{J}_{\mathbf{s}_k,k})^2_{\hat{\rho}_k}/\langle\hat{J}_{z,k}\rangle^2_{\hat{\rho}_k}$ through the choice of the $\mathbf{r}_k$ and $\mathbf{s}_k$ [16] yields the smallest possible squeezing matrix for this class of states, which is given analytically in Supplementary Equation (40).

The condition $\Xi^2[\hat{\rho}_{\mathrm{loc}},\hat{\mathbf{J}}_\mathbf{r},\hat{\mathbf{J}}_\mathbf{s}] \geq \mathbb{1}_M$ is violated already if a single mode is squeezed. If all the local states $\hat{\rho}_k$ are squeezed and therefore satisfy $\xi^2[\hat{\rho}_k,\hat{J}_{\mathbf{r}_k,k},\hat{J}_{\mathbf{s}_k,k}] < 1$, the full multimode squeezing condition $\Xi^2[\hat{\rho}_{\mathrm{loc}},\hat{\mathbf{J}}_\mathbf{r},\hat{\mathbf{J}}_\mathbf{s}] < \mathbb{1}$ is met. Such states thus lead to multiparameter sub-shot-noise sensitivities for parameters encoded locally by $\hat{\mathbf{J}}_\mathbf{r}$.

D. Nonlocal spin squeezing

1. *Local parameter encodings*

We now consider an arbitrary state $\hat{\rho}$ that may contain mode entanglement and analyze the spin squeezing matrix for local parameter encoding schemes that we found to be optimal for local squeezing. As was shown in the main manuscript, in general,

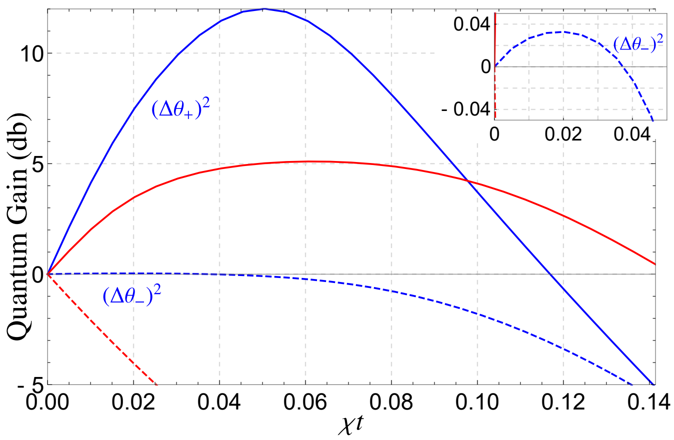

Supplementary Figure 1. Comparison between local and nonlocal squeezing with nonlocal parameter encoding. The plot shows on dB scale $10\log_{10}[(\Delta\theta_+)^2_{\mathrm{SN}}/(\Delta\theta_+)^2]$ (continuous lines) and $10\log_{10}[(\Delta\theta_-)^2_{\mathrm{SN}}/(\Delta\theta_-)^2]$ (dashed lines) for the nonlocally squeezed state $|\Psi_{\mathrm{nl}}(t)\rangle$ (blue lines) with $N = 100$ particles. The orientation $\mathbf{r}_1$ and $\mathbf{r}_2$ of the encoding generators have been locally optimized to provide the largest quantum gain. While the quantum gain for θ_+ is maximal, we observe a small gain also for θ_- at small times (see inset). The locally squeezed state $|\Psi_{\mathrm{loc}}(t)\rangle$ provides a weaker quantum gain at relevant short time scales for θ_+, and is above shot noise for θ_-, given the same choice of nonlocal Hamiltonians (red lines).

the spin squeezing matrix is no longer diagonal and its elements are described by

$$(\varXi^2[\hat{\rho}, \hat{\mathbf{J}}_{\mathbf{r}}, \hat{\mathbf{J}}_{\mathbf{s}}])_{kl} = \frac{\sqrt{N_k N_l}\mathrm{Cov}(\hat{J}_{\mathbf{s}_k,k}, \hat{J}_{\mathbf{s}_l,l})_{\hat{\rho}}}{\langle \hat{J}_{z,k}\rangle_{\hat{\rho}}\langle \hat{J}_{z,l}\rangle_{\hat{\rho}}}. \tag{43}$$

We can now understand under which conditions mode correlations further enhance the sensitivity beyond Supplementary Equation (42). We consider a linear combination of parameters, defined by the coefficients $\mathbf{n} = (n_1, \dots, n_M)^T$. According to Supplementary Equations (5) and (8), the variance of the estimation of $\mathbf{n}^T\boldsymbol{\theta} = \sum_{i=1}^M n_i\theta_i$ is given for an arbitrary quantum state $\hat{\rho}$ by

$$\mu\mathbf{n}^T\varSigma\mathbf{n} = \sum_{k,l=1}^M n_k n_l \frac{\mathrm{Cov}(\hat{J}_{\mathbf{s}_k,k}, \hat{J}_{\mathbf{s}_l,l})_{\hat{\rho}}}{\langle \hat{J}_{z,k}\rangle_{\hat{\rho}}\langle \hat{J}_{z,l}\rangle_{\hat{\rho}}} = \sum_{k,l=1}^M n_k n_l \frac{(\varXi^2[\hat{\rho}, \hat{\mathbf{J}}_{\mathbf{r}}, \hat{\mathbf{J}}_{\mathbf{s}}])_{kl}}{\sqrt{N_k N_l}}, \tag{44}$$

and in the second step, we used the definition of the spin squeezing matrix. This sum contains the weighted average of local spin-squeezing coefficients ($k = l$), in addition to the nonlocal squeezing described by covariances ($k \neq l$). It is clear that if the signs of the nonlocal squeezing terms are chosen properly and in accordance with the n_k [9], they can further enhance the sensitivity, as is illustrated by the example in the main text.

2. *Nonlocal parameter encodings*

In the main text we limited the analysis to local parameter-encoding Hamiltonians in the two spatial modes. For the locally squeezed states of the type (42), such local phase shifts and measurements are in fact optimal (recall section III C): The block diagonal structure of the matrix $\tilde{\mathcal{M}}[|\Psi_{\mathrm{loc}}(t)\rangle, \hat{\mathbf{J}}_\perp]$ ensures that the highest multiparameter sensitivity is achieved by encoding each parameter into the respective squeezed local variable via $\hat{\mathbf{H}} = \hat{\mathbf{J}}_{\mathbf{r}}$ and a collection of local observables $\hat{\mathbf{X}}_{\mathrm{opt}} = \hat{\mathbf{J}}_{\mathbf{s}}$ saturates the upper bound in Supplementary Equation (18). However, as we will see below, such local schemes are generally not optimal in the presence of mode entanglement.

We now extend our analysis of the nonlocal squeezed state $|\Psi_{\mathrm{nl}}(t)\rangle$ to nonlocal measurements and parameter-imprinting evolutions. Specifically, consider $\hat{\mathbf{H}} = (\hat{H}_1, \hat{H}_2)^T$ with $\hat{H}_1 = \frac{1}{\sqrt{2}}(\hat{J}_{\mathbf{r}_1,1} + \hat{J}_{\mathbf{r}_1,2})$ and $\hat{H}_2 = \frac{1}{\sqrt{2}}(\hat{J}_{\mathbf{r}_2,1} - \hat{J}_{\mathbf{r}_2,2})$, where $\mathbf{r}_1, \mathbf{r}_2$ are two orthonormal vectors in the xy plane, as well as the measurement observables $\hat{\mathbf{X}} = (\hat{X}_1, \hat{X}_2)^T$ with $\hat{X}_1 = \frac{1}{\sqrt{2}}(\hat{J}_{\mathbf{r}_2,1} + \hat{J}_{\mathbf{r}_2,2})$ and $\hat{X}_2 = \frac{1}{\sqrt{2}}(\hat{J}_{\mathbf{r}_1,2} - \hat{J}_{\mathbf{r}_1,1})$. This scheme describes a nonlocal encoding of two parameters $\boldsymbol{\theta} = (\theta_+, \theta_-)^T$ by the evolution $\hat{U}(\boldsymbol{\theta}) = \exp(-i\hat{H}_1\theta_+ - i\hat{H}_2\theta_-)$. We obtain the squeezing matrix

$$\varXi^2[|\Psi_{\mathrm{nl}}(t)\rangle, \hat{\mathbf{H}}, \hat{\mathbf{X}}] = \begin{pmatrix} \xi_+^2 & 0 \\ 0 & \xi_-^2 \end{pmatrix}. \tag{45}$$

After an optimization over $\mathbf{r}_1$ and $\mathbf{r}_2$, the eigenvalue ξ_+^2 coincides with the single-parameter spin-squeezing coefficient for the full ensemble of N atoms: $\xi_+^2 = \min_{\mathbf{r}_1,\mathbf{r}_2} N(\Delta\hat{J}_{\mathbf{r}_2})^2_{|\Psi_{\mathrm{nl}}(t)\rangle}/|\langle[\hat{J}_{\mathbf{r}_1},\hat{J}_{\mathbf{r}_2}]\rangle_{|\Psi_{\mathrm{nl}}(t)\rangle}|^2$ with $\hat{J}_{\mathbf{r}} = \hat{J}_{\mathbf{r},1} + \hat{J}_{\mathbf{r},2}$, and indicates significant quantum enhancements (blue line in Supplementary Figure 1). The quantum gain expressed by it can be achieved through the collective evolution and measurement operators $\hat{H}_1$ and $\hat{X}_1$. In contrast, the gain ξ_-^2 is accessible only by local measurements on the two spin ensembles. We notice that sub-shot-noise measurements of one parameter do not necessarily imply a reduced sensitivity below the classical limit for the other: At short times, the sensitivity of θ_- remains close to the shot-noise limit and even slightly undercuts it for very small χt. For comparison, the weaker sensitivity of the locally squeezed state $|\Psi_{\mathrm{loc}}(t)\rangle$ is shown (red lines).

For the nonlocally squeezed state, the choice of phase-imprinting generators $\hat{\mathbf{H}}$ defined above is optimal: The eigenvalues of $\mathcal{M}_{\mathrm{opt}}[|\Psi_{nl}(t)\rangle, \mathbf{H}, \hat{\mathbf{J}}_\perp]$ are maximized by this choice of $\hat{\mathbf{H}}$. Since the shot-noise matrix $F_{\mathrm{SN}}[\hat{\mathbf{H}}]$ is diagonal, the same $\hat{\mathbf{H}}$ minimize also the eigenvalues of the squeezing matrix $\mathit{\Xi}^2_{\mathrm{opt}}[|\Psi_{\mathrm{nl}}(t)\rangle, \hat{\mathbf{H}}, \hat{\mathbf{J}}_\perp]$. Furthermore, the $\hat{\mathbf{X}}$ satisfy the optimality condition Supplementary Equation (20), leading to $\mathit{\Xi}^2[|\Psi_{\mathrm{nl}}(t)\rangle, \hat{\mathbf{H}}, \hat{\mathbf{X}}] = \mathit{\Xi}^2_{\mathrm{opt}}[|\Psi_{\mathrm{nl}}(t)\rangle, \hat{\mathbf{H}}, \hat{\mathbf{J}}_\perp]$, where the optimal squeezing matrix was defined in Supplementary Equation (37). Moreover, additional $M \times M$ orthogonal transformations $\hat{\mathbf{H}}' = V\hat{\mathbf{H}}$ can be used to modify the parameter-encoding evolution with the effect of changing the eigenvectors of $\mathit{\Xi}^2_{\mathrm{opt}}[|\Psi_{\mathrm{nl}}(t)\rangle, \hat{\mathbf{H}}, \hat{\mathbf{J}}_\perp]$ without changing the eigenvalues. We recall that such transformations have no impact on the optimality of the measurement observables, due to the freedom provided by the matrix T in Supplementary Equation (20).

IV. SUPPLEMENTARY NOTE 4: CONTINUOUS-VARIABLE SQUEEZING MATRIX

A. Equivalence to the squeezing condition by Simon *et al.*

We show that in the context of continuous-variable systems and considering quadrature observables $\hat{\mathbf{q}}$, the squeezing condition, i.e., any violation of

$$\mathit{\Xi}^2[\hat{\rho}, \hat{\mathbf{H}}, \hat{\mathbf{X}}] \geq \mathbb{1}_M, \tag{46}$$

becomes equivalent to

$$\lambda_{\min}(\mathit{\Gamma}[\hat{\rho}, \hat{\mathbf{q}}]) < \frac{1}{4}, \tag{47}$$

which was proposed by Simon *et al.* in Supplementary Reference [17].

Assume that (46) is violated by the matrix $\mathit{\Xi}^2[\hat{\rho}, \hat{\mathbf{H}}, \hat{\mathbf{X}}]$ for some $\hat{\mathbf{H}} = R\hat{\mathbf{q}}$ and $\hat{\mathbf{X}} = S\hat{\mathbf{q}}$. Since $\mathit{\Xi}^2_{\mathrm{opt}}[\hat{\rho}, \hat{\mathbf{H}}, \hat{\mathbf{q}}] \leq \mathit{\Xi}^2[\hat{\rho}, \hat{\mathbf{H}}, \hat{\mathbf{X}}]$ for all $\hat{\mathbf{X}}$, this implies that also $\mathit{\Xi}^2_{\mathrm{opt}}[\hat{\rho}, \hat{\mathbf{H}}, \hat{\mathbf{q}}]$ violates condition (46). This is equivalent to $\lambda_{\min}(\mathit{\Xi}^2_{\mathrm{opt}}[\hat{\rho}, \hat{\mathbf{H}}, \hat{\mathbf{q}}]) < 1$. Because R is an orthogonal projection onto an M-dimensional subspace, the matrix $\mathit{\Xi}^2_{\mathrm{opt}}[\hat{\rho}, \hat{\mathbf{H}}, \hat{\mathbf{q}}]$ is a compression of the $2M \times 2M$ matrix $4\mathit{\Omega}^T\mathit{\Gamma}[\hat{\rho}, \hat{\mathbf{q}}]\mathit{\Omega}$. By the inclusion principle [18], we obtain $1 > \lambda_{\min}(\mathit{\Xi}^2_{\mathrm{opt}}[\hat{\rho}, \hat{\mathbf{H}}, \hat{\mathbf{q}}]) \geq \lambda_{\min}(4\mathit{\Omega}^T\mathit{\Gamma}[\hat{\rho}, \hat{\mathbf{q}}]\mathit{\Omega}) = 4\lambda_{\min}(\mathit{\Gamma}[\hat{\rho}, \hat{\mathbf{q}}])$, and we used that $\mathit{\Omega}$ is an orthogonal matrix. Hence, $\lambda_{\min}(\mathit{\Gamma}[\hat{\rho}, \hat{\mathbf{q}}]) < 1/4$.

Conversely, assume that $\lambda_{\min}(\mathit{\Gamma}[\hat{\rho}, \hat{\mathbf{q}}]) < 1/4$ holds, then there exists an R, such that $\lambda_{\min}(\mathit{\Xi}^2_{\mathrm{opt}}[\hat{\rho}, \hat{\mathbf{H}}, \hat{\mathbf{q}}]) = \lambda_{\min}(4\mathit{\Omega}^T\mathit{\Gamma}[\hat{\rho}, \hat{\mathbf{q}}]\mathit{\Omega}) = 4\lambda_{\min}(\mathit{\Gamma}[\hat{\rho}, \hat{\mathbf{q}}])$. This yields $\lambda_{\min}(\mathit{\Xi}^2_{\mathrm{opt}}[\hat{\rho}, \hat{\mathbf{H}}, \hat{\mathbf{q}}]) < 1$ and hence the squeezing condition (46) can be violated for some $\hat{\mathbf{X}}$.

B. Minimizing the squeezing matrix

Let $\lambda'_1 \leq \cdots \leq \lambda'_M$ and $\lambda_1 \leq \cdots \leq \lambda_{2M}$ denote the eigenvalues of $\mathit{\Xi}^2_{\mathrm{opt}}[\hat{\rho}, \hat{\mathbf{H}}]$ and $4\mathit{\Omega}^T\mathit{\Gamma}[\hat{\rho}, \hat{\mathbf{q}}]\mathit{\Omega}$, respectively. The inclusion principle yields $\lambda'_k \leq \lambda_k$ for all $k = 1, \ldots, M$. The minimum spectrum is reached when $\lambda'_k = \lambda_k$ holds for all $k = 1, \ldots, M$. To achieve this, we choose $\hat{\mathbf{H}}_{\mathrm{opt}} = R_{\mathrm{opt}}\hat{\mathbf{q}}$, by picking the rows of R_{opt} as $\mathbf{r}_k = \boldsymbol{\lambda}_k$ for $k = 1, \ldots, M$, where the $\boldsymbol{\lambda}_k$ are the eigenvectors of $4\mathit{\Omega}^T\mathit{\Gamma}[\hat{\rho}, \hat{\mathbf{q}}]\mathit{\Omega}$ with eigenvalue λ_k.

For the squeezed vacuum state, the eigenvectors $\boldsymbol{\lambda}_k$ form the symplectic orthogonal matrix $O\mathit{\Omega}$, where the columns are ordered in pairs acting on the same mode. The projector P_M then selects only the squeezed quadratures from each mode, thereby realizing $R_{\mathrm{opt}} = P_M O\mathit{\Omega}$ as described above. From Supplementary Equation (20) we find the optimal measurement operators as $\hat{\mathbf{X}}_{\mathrm{opt}} = TP_M O\mathit{\Omega}\mathit{\Omega}^T\mathit{\Gamma}[|\Psi_0\rangle, \hat{\mathbf{q}}]^{-1}\hat{\mathbf{q}} = TP_M O\mathit{\Gamma}[|\Psi_0\rangle, \hat{\mathbf{q}}]^{-1}O^T O\hat{\mathbf{q}} = 4TP_M \bigoplus_{k=1}^M \mathrm{diag}(e^{2r_k}, e^{-2r_k})O\hat{\mathbf{q}}$, and we obtain $\hat{\mathbf{X}}_{\mathrm{opt}} = P_M O\hat{\mathbf{q}}$ by choosing $T = \frac{1}{4}\mathrm{diag}(e^{2r_1}, \ldots, e^{2r_M})$. This choice leads to $C[|\Psi_0\rangle, \hat{\mathbf{H}}, \hat{\mathbf{X}}] = \frac{1}{2}S\mathit{\Omega}R^T = \frac{1}{2}P_M O\mathit{\Omega}\mathit{\Omega}^T O^T P_M^T = \frac{1}{2}\mathbb{1}_M$.

C. Changing the basis of the squeezing matrix by passive transformations

Let us consider a fixed family of encoding Hamiltonians with $\hat{\mathbf{H}} = P_M O\mathit{\Omega}\hat{\mathbf{q}}$. Any squeezed vacuum state can be expressed as $\hat{U}_V|\Psi_0\rangle$. We obtain $\mathit{\Gamma}[\hat{U}_V|\Psi_0\rangle, \hat{\mathbf{q}}] = V^T\mathit{\Gamma}[|\Psi_0\rangle, \hat{\mathbf{q}}]V$, where V is the symplectic orthogonal matrix that describes

the passive transformation $\hat{U}_V$. For an optimal choice of measurement operators $\hat{\mathbf{X}}$, the state $\hat{U}_V|\Psi_0\rangle$ leads to the squeezing matrix $\Xi^2_{\rm opt}[\hat{U}_V|\Psi_0\rangle, \hat{\mathbf{H}}] = 4P_M O V^T \varGamma[|\Psi_0\rangle, \hat{\mathbf{q}}] V O^T P_M^T$. Let Y be the orthogonal symplectic matrix that yields $4\varGamma[|\Psi_0\rangle, \hat{\mathbf{q}}] = Y^T \bigoplus_{k=1}^M \mathrm{diag}(e^{2r_k}, e^{-2r_k}) Y$. Choosing $V = Y^T W O$, we obtain $\Xi^2_{\rm opt}[\hat{U}_V|\Psi_0\rangle, \hat{\mathbf{H}}] = P_M W^T \bigoplus_{k=1}^M \mathrm{diag}(e^{2r_k}, e^{-2r_k}) W P_M^T$. The symplectic orthogonal matrix W can now be chosen such that

$$\Xi^2_{\rm opt}[\hat{U}_V|\Psi_0\rangle, \hat{\mathbf{H}}] = \sum_{k=1}^M e^{-2r_k} \mathbf{n}_k \mathbf{n}_k^T, \tag{48}$$

where $\{\mathbf{n}_k\}_{k=1}^M$ is an arbitrary basis of $\mathbb{R}^M$.

For clarity, let us explicitly construct the matrix W that achieves this. We represent the projector as $P_M = \sum_{i=1}^M \mathbf{e}_i \mathbf{f}_{2i}^T$, where $\{\mathbf{e}_i\}_{i=1}^M$ and $\{\mathbf{f}_i\}_{i=1}^{2M}$ represent canonical bases of $\mathbb{R}^M$ and $\mathbb{R}^{2M}$, respectively. We define $\mathbf{m}_{2i} = (0, n_{i1}, \dots, 0, n_{iM})^T$ and $\mathbf{m}_{2i-1} = (n_{i1}, 0, \dots, n_{iM}, 0)^T$ for $i = 1, \dots, M$. The $\{\mathbf{m}_i\}_{i=1}^{2M}$ form a basis of $\mathbb{R}^{2M}$. By choosing $W = \sum_{i=1}^{2M} \mathbf{m}_i \mathbf{f}_i^T$, we obtain the squeezing matrix provided in Supplementary Equation (48). By construction the matrix W is orthogonal. By writing $\varOmega = \sum_{i=1}^M (\mathbf{f}_{2i-1}\mathbf{f}_{2i}^T - \mathbf{f}_{2i}\mathbf{f}_{2i-1}^T)$ and making use of $\sum_{i=1}^M n_{ki} n_{li} = \mathbf{n}_k^T \mathbf{n}_l = \delta_{kl}$, it is possible to demonstrate explicitly that $W \varOmega W^T = \varOmega$ and thus W is symplectic. Finally, let us consider the optimal measurement observables $\hat{\mathbf{X}} = T P_M O \varGamma[\hat{U}_V|\Psi_0\rangle, \hat{\mathbf{q}}]^{-1} \hat{\mathbf{q}} = 4 T P_M W^T \bigoplus_{k=1}^M \mathrm{diag}(e^{-2r_k}, e^{2r_k}) W O \hat{\mathbf{q}}$. Choosing $T = \frac{1}{4}\mathrm{diag}(e^{2r_1}, \dots, e^{2r_M})$ yields $\hat{\mathbf{X}} = P_M O \hat{\mathbf{q}}$. It is interesting to notice that the optimal measurement observables are thus independent of the basis that is chosen by W. Instead they depend only on O, i.e., the phase-imprinting generators $\hat{\mathbf{H}}$.

D. Optimality of squeezed vacuum states

In Supplementary Reference [19] the intuition about the optimality of squeezed vacuum states for single-parameter estimation with continuous-variable systems [20] was confirmed by a rigorous demonstration. We now show that analogous results hold for multiparameter estimation problems, where for the optimization we distinguish between the two cases discussed in the previous section.

Let us first discuss the optimization of the spectrum of the quantum Fisher matrix $F_Q[\hat{\rho}, \hat{\mathbf{H}}]$. For mode-separable probe states the upper sensitivity limit is given by the block-diagonal covariance matrix $\max_{\hat{\rho}_{\rm m-sep}} F_Q[\hat{\rho}_{\rm m-sep}, \hat{\mathbf{H}}] = 4 \bigoplus_{k=1}^M \max_{|\psi_k\rangle} (\Delta \hat{H}_k)^2_{|\psi_k\rangle}$ and the convexity of F_Q allows us to limit the optimization to pure states [9]. Since each $\hat{H}_k$ is a local quadrature operator, the local variances satisfy $4(\Delta \hat{H}_k)^2_{|\Psi_k\rangle} \leq 2N_k + 1 + 2|\langle \hat{a}_k \hat{a}_k \rangle_{|\psi_k\rangle}|$ with $N_k = \langle \hat{a}^\dagger \hat{a} \rangle_{|\psi_k\rangle}$. This bound can be derived by taking the larger of the two eigenvalues of the covariance matrix $\varGamma[|\psi_k\rangle, (\hat{x}_k, \hat{p}_k)^T]$, and by setting all mean values to zero (which can only increase the covariance matrix). From the Cauchy-Schwarz inequality we obtain $|\langle \hat{a}_k \hat{a}_k \rangle_{|\psi_k\rangle}|^2 \leq \langle \hat{a}_k^\dagger \hat{a}_k \rangle_{|\psi_k\rangle} \langle \hat{a}_k \hat{a}_k^\dagger \rangle_{|\psi_k\rangle} = N_k(N_k + 1)$. This finally yields $\max_{|\psi_k\rangle} 4(\Delta \hat{H}_k)^2_{|\psi_k\rangle} = 2N_k + 1 + 2\sqrt{N_k(N_k+1)}$. This upper limit is saturated by a non-displaced squeezed vacuum state, as can be easily verified using $2N_k + 1 = \cosh 2r_k$ and $\sinh 2r_k = \pm 2\sqrt{N_k(N_k+1)}$.

Next, we consider the estimation of a specific linear combination of parameters, defined by the coefficient vector $\mathbf{n} \in \mathbb{R}^M$. The variance $\mu \mathbf{n}^T \varSigma \mathbf{n} = (\mathbf{n}^T F_Q[\hat{\rho}, \hat{\mathbf{H}}] \mathbf{n})^{-1}$ is minimized by a pure state with $F_Q[|\Psi\rangle, \hat{\mathbf{H}}] = 4\varGamma[|\Psi\rangle, \hat{\mathbf{H}}]$. Assuming $\mathbf{n}$ to be normalized to one, the sensitivity limit is given by the largest eigenvalue of the $M \times M$ covariance matrix $\max_{|\Psi\rangle} 4\varGamma[|\Psi\rangle, \hat{\mathbf{H}}]$, where $\hat{\mathbf{H}} = P_M O \hat{\mathbf{q}}$. It is achieved when $\mathbf{n}$ represents the corresponding eigenvector. Since O is a canonical transformation, each eigenvalue of $\varGamma[|\Psi\rangle, \hat{\mathbf{H}}]$ corresponds to the variance of some quadrature observable that is constructed as a linear combination of the original $\hat{\mathbf{q}}$ and follows the same commutation relations. Following the arguments from above, we obtain the bound $\lambda_{\max}(4\varGamma[|\Psi\rangle, \hat{\mathbf{H}}]) \leq 2N + 1 + 2\sqrt{N(N+1)}$, which is again saturated by squeezed vacuum states. Here $N = \sum_{k=1}^M N_k$ is the total number of particles.

This extends the results of Supplementary Reference [19] and demonstrates the optimality of squeezed vacuum also in the multiparameter case. Similar strategies are optimal also for more general multimode passive Gaussian channels that encode a single [21] or multiple phases [22]. If transformations beyond displacements are considered, however, the preparation of the optimal state is no longer independent of the values of the unknown phases and thus requires adaptive methods.

SUPPLEMENTARY REFERENCES

[1] Lehmann, E. L. & Casella, G. *Theory of Point Estimation*, (Springer, New York, 1998).

[2] Kay, S. M. *Fundamentals of Statistical Signal Processing: Estimation Theory* (Englewood Cliffs, NJ, 1993).

[3] Chipman, J. S. On Least Squares with Insufficient Observations, J. Am. Stat. Assoc. **59**, 1078–1111 (1964).

[4] Pečarić, J. E., Puntanen, S. & Styan, G. P. H., Some further matrix extensions of the Cauchy-Schwarz and Kantorovich inequalities, with some statistical applications, Linear Algebra Appl. **237-238**, 455–476 (1996).

[5] Gessner, M., Enhancement of the metrological sensitivity limit through knowledge of the average energy, Phys. Rev. A **100**, 032114 (2019).
[6] Pezzè, L., Ciampini, M. A., Spagnolo, N., Humphreys, P. C., Datta, A., Walmsley, I. A., Barbieri, M., Sciarrino, F. & Smerzi, A. Optimal Measurements for Simultaneous Quantum Estimation of Multiple Phases, Phys. Rev. Lett. **119**, 130504 (2017).
[7] Helstrom, C. W. *Quantum Detection and Estimation Theory* (Academic Press, New York, 1976).
[8] Gessner, M., Smerzi, A. & Pezzè, L. Metrological Nonlinear Squeezing Parameter, Phys. Rev. Lett. **122**, 090503 (2019).
[9] Gessner, M., Pezzè, L. & Smerzi, A. Sensitivity Bounds for Multiparameter Quantum Metrology, Phys. Rev. Lett. **121**, 130503 (2018).
[10] Ferraro, A., Olivares, S. & Paris, M. G. A. *Gaussian states in continuous variable quantum information* (Bibliopolis, Napoli, 2005), Preprint available at http://arxiv.org/abs/quant-ph/0503237.
[11] Wang, X., Hiroshima, T., Tomita, A. & Hayashi, M. Quantum information with Gaussian states, Phys. Rep. **448**, 1-111 (2007).
[12] Weedbrook, C., Pirandola, S., García-Patróon, R., Cerf, N. J., Ralph, T. C., Shapiro, J. H. & Lloyd, S., Gaussian quantum information, Rev. Mod. Phys. **84**, 621 (2012).
[13] Wineland, D. J., Bollinger, J. J., Itano, W. M., Moore, F. L. & Heinzen, D. J. Spin squeezing and reduced quantum noise in spectroscopy, Phys. Rev. A **46**, R6797 (1992).
[14] Wineland, D. J., Bollinger, J. J., Itano, W. M. & Heinzen, D. J. Squeezed atomic states and projection noise in spectroscopy, Phys. Rev. A **50**, 67 (1994).
[15] Pezzè, L., Smerzi, A., Oberthaler, M. K., Schmied, R. & Treutlein, P. Quantum metrology with nonclassical states of atomic ensembles, Rev. Mod. Phys. **90**, 035005 (2018).
[16] Ma, J., Wang, X., Sun, C. & Nori, F. Quantum spin squeezing, Phys. Rep. **509**, 89 (2011).
[17] Simon, R., Mukunda, N. & Dutta, B. Quantum-noise matrix for multimode systems: $U(n)$ invariance, squeezing, and normal forms, Phys. Rev. A **49**, 1567 (1994).
[18] Bernstein, D. S. Matrix Mathematics, (Princeton University Press, Princeton, NJ, 2009).
[19] Lang, M. D. & Caves, C. M. Optimal Quantum-Enhanced Interferometry Using a Laser Power Source, Phys. Rev. Lett. **111**, 173601 (2013).
[20] Joo, J., Munro, W. J. & Spiller, T. P. Quantum Metrology with Entangled Coherent States, Phys. Rev. Lett. **107**, 083601 (2011).
[21] Matsubara, T., Facchi, P., Giovannetti, V. & Yuasa, K., Optimal Gaussian metrology for generic multimode interferometric circuit, New J. Phys. **21**, 033014 (2019).
[22] Oh, C., Lee, C., Hyung Lie, S. & Jeong, H. Optimal Distributed Gaussian Sensing, Phys. Rev. Research **2**, 023030 (2020).
